# High-Protein Intake Impact on Human and Animal Kidney Function Across the Lifespan: Clinical Evidence and Emerging Concepts

**DOI:** 10.3390/nu18193209

**Published:** 2026-09-29

**Authors:** Joanna Jacuńska, Aleksandra Bruciak, Jakub Wilk, Wojciech Grodzki, Kinga Musiał

**Affiliations:** 1 Students’ Scientific Association, Department of Pediatric Nephrology, Wroclaw Medical University, Borowska 213, 50-556 Wroclaw, Poland; 2 Department of Pediatric Nephrology, Wroclaw Medical University, Borowska 213, 50-556 Wroclaw, Poland

**Keywords:** animal protein, children, chronic kidney disease, estimated glomerular filtration rate, hyperfiltration, high-protein intake, plant protein

## Abstract

Background/Objectives: High-protein diets have gained increasing popularity in recent years. This trend has raised concerns regarding its impact on renal function, particularly among individuals with chronic kidney disease (CKD). The aim of this narrative review is to summarize current evidence on the effect of high-protein intake on kidney function. An analysis across species and age groups takes into account both theoretical and clinical perspectives. Methods: A literature search was conducted in PubMed, Scopus, and Web of Science to identify experimental and animal studies, as well as data concerning adult and pediatric populations, with special focus on those with CKD. Results: Experimental studies confirm protein-triggered adaptive increase in intraglomerular pressure and subsequent hyperfiltration, yet without any long-term negative effect on a healthy kidney. Human adult studies indicate that high-protein dietary content may accelerate the progression of CKD. Conversely, in older individuals, high protein intake combined with plant-rich diet appears to protect against sarcopenia and is associated with increased longevity. In children with CKD, protein intake should be adjusted according to growth requirements. In both pediatric and general populations, increasing the proportion of plant-based protein relative to animal protein may improve the health outcomes, although evidence on kidney function protection is insufficient. Conclusions: A balance should be maintained between excessive protein intake, which may aggravate existing dysfunctions, and overly restrictive protein consumption, which may lead to sarcopenia in older adults and impaired growth/development in children. A personalized approach is essential, taking into account age, kidney function/CKD stage, comorbidities, and, in the pediatric population, growth rate and developmental needs.

## 1. Introduction

High-protein diets (HPD) have gained considerable interest in recent years due to their reported benefits in weight reduction, muscle preservation, and metabolic control. Consequently, societies all around the world are distancing themselves from traditional cuisines. Data collected from the last decade have shown a decreasing adherence to plant-based dietary patterns, even in the Mediterranean basin [1,2,3].

Meanwhile, there is an increasing trend of protein consumption in the American society [4,5]. The term “ketogenic diet”, meaning 25% of calories obtained from protein, was the most searched dietary pattern in the US in 2020 [6]. Even though the efficiency of ketogenic diet in treating hypertension or diabetes has not been confirmed, the market for “keto” products was worth over $9.57 billion in 2019 [7].

The recommended daily protein intake is 0.8 g/kg/day for a healthy adult. High protein intake exceeds this value by 25% (>1.0 g/kg/day). However, when the routine dietary content of protein in Western countries (1.0–1.4 g/kg/day in adults) is confronted with this definition, pushing the “high-protein” boundaries above the 1.5 g/kg/day by some authors seems justified.

Since protein metabolism is tightly connected to renal function, the latter should respond to excessive protein intake. The kidneys play a central role in nitrogen excretion, acid–base balance, and the regulation of the glomerular filtration rate (GFR), in connection with dietary protein load [8,9]. A single increased protein intake can transiently elevate the GFR, a phenomenon known as glomerular hyperfiltration. The consequences of repetitive dietary protein overload in healthy subjects are not fully known [10,11]. Moreover, emerging evidence suggests that individual responses to dietary protein may differ according to genetic and microbiome-related factors [8].

Another major determinant of renal response to protein intake is previous kidney damage. Chronic kidney disease (CKD) is a perfect example of complex relations between protein load and renal function, depending on the amount and quality of protein consumed, baseline renal function, comorbidities, and metabolic adaptation [8,11,12].

This review aims to summarize the current evidence on dietary protein intake and its impact on kidney function across species and age groups. It highlights physiological mechanisms, data from experimental models, clinical findings from observational studies in adults and children, as well as data concerning patients with chronic kidney disease of all age groups. Integration of recent advances and guidelines should provide clinicians and researchers with an up-to-date practical framework on how to balance protein adequacy and renal protection in healthy subjects and patients with CKD.

## 2. Materials and Methods

### 2.1. Literature Search Strategy

This narrative review is based on a literature search conducted in PubMed, Scopus, and Web of Science Core Collection. We aimed to identify studies examining the relationship between dietary protein intake and kidney function on experimental level of animal studies, in healthy humans, and in patients with chronic kidney disease (CKD), including children. The search covered records available from 1960 until May 2026 (Figure 1).

The search strategy combined controlled vocabulary and free-text terms related to dietary protein intake (“high-protein diet”, “protein intake”, “dietary protein”, “low-protein diet”), kidney outcomes (“chronic kidney disease”, “CKD”, “glomerular filtration rate”, “eGFR”, “hyperfiltration”, “albuminuria”), and study populations (“adult”, “older adult”, “children”, “pediatric”, “animal model”). Searches were primarily performed within titles, abstracts, and keywords.

Original studies, randomized controlled trials, cohort studies, systematic reviews, meta-analyses, and relevant clinical practice guidelines published in English were considered eligible. Reference lists of key publications were additionally screened to identify seminal studies that were not retrieved during the electronic database search.

### 2.2. Data Sources

Studies were categorized according to their primary focus into the following predefined themes: physiological mechanisms linking protein intake to kidney function, experimental and animal studies, observational and interventional studies in adults, pediatric evidence, clinical nutrition guidelines, and practical implications for CKD management.

### 2.3. Study Selection and Eligibility Criteria

Publications were assessed by two independent reviewers to exclude irrelevant studies. Then, a full-text review was performed to confirm eligibility. Inclusion criteria were as follows: original research articles, systematic reviews, or meta-analyses investigating dietary protein intake and renal outcomes; studies involving human participants (adults or children) or validated animal models of CKD; quantitative or qualitative assessment of kidney function (e.g., eGFR, serum creatinine, albuminuria, kidney histology); articles published in peer-reviewed journals and available in English. Exclusion criteria included: case reports, editorials, or narrative reviews lacking original data; studies evaluating short-term metabolic effects without renal outcome measures; papers focusing on acute kidney injury (AKI); duplicate publications or overlapping datasets.

### 2.4. Data Extraction and Categorization

Data from each eligible study were extracted using a standardized form capturing: author(s) and year of publication; population (adults, pediatric, or animal); study design (observational, RCT, mechanistic); sample size (cohort vs. small groups) and protein intake level (low, moderate, high); primary renal outcomes (decreased/normal/increased eGFR); key findings and clinical implications. Studies were subsequently grouped into five thematic categories for narrative synthesis of dietary protein intake: physiological and mechanistic insights; animal and experimental models; general healthy population and adults with pre-dialysis CKD; distinctive features of pediatric development and pre-dialysis CKD; patients on chronic dialysis.

## 3. Physiological Mechanisms Linking Protein Intake to Kidney Function

Dietary protein has a significant impact on kidney physiology via hemodynamic and metabolic effects. The kidneys play a central role in nitrogen excretion, acid–base regulation, and maintenance of the glomerular filtration rate (GFR). The recommended amount of protein intake for an average adult person with minimal physical activity has been set to 0.8 g per kg of body mass per day [13]. When protein intake increases to over 1.5 g per kg, a complex set of adaptive mechanisms is activated to manage the additional nitrogenous and acid load [14]. Chronic high protein intake may exhaust these compensatory reactions. Triggering morphological and functional changes may contribute to renal injury in those with already decreased nephron mass or established CKD [8,15].

### 3.1. Hemodynamic Stress and Glomerular Hyperfiltration

The physiological answer to a high-protein meal is a transient rise in GFR. This response is mediated by a complex interplay between amino acid-induced vasodilation, increased renal plasma flow, and the tubulo-glomerular feedback mechanism [16]. Experimental studies have demonstrated that amino acid loading stimulates afferent arteriolar dilation via nitric oxide and prostaglandin pathways, resulting in increased intraglomerular pressure. The latter triggers hyperfiltration lasting for a few hours, subsequent to protein intake.

Transient hyperfiltration after protein ingestion is a physiological response to adaptive processes in normal kidneys. Data in humans show that those who consume high-protein diets have increased single-nephron GFR and renal plasma flow, when compared to those consuming moderate-protein diets [8,15]. Prolonged increase in intraglomerular pressure may trigger mechanical forces on the filtration barrier of a healthy kidney, resulting in hyperfiltration without long-term negative sequelae [8,15]. Therefore, the originally intact kidney function remains quite resistant to potential noxious effects of hyperfiltration [8,15]. However, the long-term increase in intraglomerular pressure within the previously damaged glomerulus may exacerbate the already existing dysfunction, aggravating injury or adding to chronic kidney disease progression [8,15].

### 3.2. Hormonal and Metabolic Mediators

Protein-induced hyperfiltration is also modulated by endocrine signals, notably insulin-like growth factor-1 (IGF-1), glucagon, and vasopressin. Elevated IGF-1 levels, typical for high protein intake, stimulate renal hypertrophy and glomerular enlargement [8]. Glucagon contributes by promoting renal vasodilation and enhancing intraglomerular pressure. Vasopressin, secreted in response to dietary protein and osmotic load, exerts additional hemodynamic stress and has been implicated in the pathogenesis of cystic and hypertensive kidney disease [17].

Moreover, high-protein load increases renal oxygen demand and mitochondrial workload, predisposing the kidney to oxidative stress and reactive oxygen species (ROS) production. Experimental evidence shows that chronic oxidative stress activates TGF-β and mTOR signaling, leading to interstitial fibrosis and tubular atrophy [15].

### 3.3. Acid Load, Phosphate Burden, and Endothelial Dysfunction

Animal-derived proteins, particularly those rich in sulfur-containing amino acids (methionine, cysteine), generate a high acid load. It must be buffered by renal ammoniagenesis. Chronic metabolic acidification contributes to tubular injury, promotes endothelin-1 release, and impairs endothelial nitric oxide availability, thus leading to endothelial dysfunction and renal microvascular damage [18,19].

Plant foods, such as beans, nuts, peas and legumes, remain rich in protein and phosphorus. The latter is mostly stored in phytate and phytic acid, degraded by phytase. People lack this enzyme, therefore phosphate bioavailability from plant products is lower than from the animal ones [20]. On the contrary, animal foods preserve phosphorus in organic form, bound to intracellular compartments and molecules. Such reservoir reveals changeable (40–60%) bioavailability in the intestinal tract. Thus, ingesting the same amount of protein may result in lower absorption of phosphorus from plant-based sources [21].

Additionally, dietary phosphate load depends strongly on the content of food additives and preservatives. Highly processed foods might boost extra phosphorus intake even up to 1000 mg/day [22,23,24,25]. Confrontation with a phosphorus mean demand established at 500–700 mg/day makes the actual ingestion doubled [26]. Unfortunately, excessive delivery of inorganic phosphate can exacerbate hyperphosphatemia, vascular calcification, and secondary hyperparathyroidism in patients with advanced stages of CKD [23,27]. These metabolic perturbations reinforce the importance of considering not only the quantity, but also the source and quality of dietary protein. In practice, moderate protein intake (0.9–1.0 g/kg/day) with avoidance of processed and convenience foods should secure a safe phosphate daily dose.

### 3.4. Role of Protein Source: Animal vs. Plant-Based Proteins

Emerging evidence suggests that plant-based proteins exert less renal stress than animal proteins. Plant sources typically produce a lower acid load, contain more potassium and fiber, and are accompanied by beneficial bioactive compounds such as polyphenols and isoflavones [28,29]. Moreover, substituting a portion of animal protein with plant protein is associated with lower albuminuria, reduced oxidative stress, and slower CKD progression [30,31]. It also lowers total cholesterol level, ApoB, LDL cholesterol and triglycerides [30].

Mechanistically, plant proteins trigger glomerular hyperfiltration, as well as stimulation of IGF-1 and vasopressin signaling pathways, to a lesser extent compared to animal proteins [17]. In addition, lower bioavailability of phosphorus in plant foods due to phytates may also blunt phosphate-induced renal damage [32].

### 3.5. Adaptive Versus Maladaptive Responses

It is important to recognize that not all renal adaptive reactions to higher protein intake are deleterious. In individuals with normal kidney function, hyperfiltration can serve as a physiological reserve allowing for efficient nitrogen excretion. However, in CKD, where nephron number and/or functional reserve are diminished, the same mechanisms become maladaptive, accelerating disease progression. Human cohort studies have confirmed that the impact of dietary protein on renal outcomes is modulated by baseline kidney function, with detrimental effects observed primarily in those with eGFR < 60 mL/min/1.73 m^2^ [33].

Likewise, animal studies confirm progression of kidney structure changes, owing to sustained high-protein feeding, in models with pre-existing nephron reduction [34].

### 3.6. Dietary Protein Influence on Gut Microbiota

Gut dysbiosis accompanies chronic kidney disease, favoring bacterial translocation and leakage of uremic toxins into the bloodstream [35,36,37]. The latter adds to the microinflammation already present in CKD conditions.

A study conducted in mice proved low-protein high-fiber diet acted in an anti-inflammatory way by reducing CD4+ and Th17+ subpopulations [38]. Likewise, low protein intake (0.6 g/kg/day), combined with inulin supplementation, improved QoL among patients with CKD stage 3–4 [39,40,41]. Although no impact on eGFR was recorded, better metabolic control, reduced microinflammation, and improved physical performance were noticed [39,40,41].

## 4. Animal and Experimental Models

### 4.1. Overview

Animal studies provide mechanistic insights into how exposure to high protein intake affects kidney structure and function. These models demonstrate that sustained protein overload leads to glomerular hypertrophy, hyperfiltration, and progressive sclerosis.

In rodent models, high-protein feeding usually consists of 10 g/kg of body weight/day, which is out of proportion to any high protein content in humans. In Wistar rats, 12 weeks of a high-protein diet increased kidney weight by ~20%, expanded mesangial area by ~30%, and enlarged glomerular tuft size [42]. More acidic urine and a marked fall in urinary citrate concentration followed, indicating both glomerular and tubular stress despite largely unchanged plasma biochemistry [42]. Moreover, a decrease in urinary citrate concentration might create a favorable environment for kidney stone formation, additionally aggravating the risk of kidney damage. In female Sprague–Dawley rats, long-term provision of a diet supplying 35% of energy from protein led to sustained hyperfiltration, approximately three-fold higher proteinuria, glomerular hypertrophy, glomerulosclerosis and tubulointerstitial fibrosis. Simultaneously, activation of pro-fibrotic and pro-inflammatory mediators such as TGF-β1 and the MCP-1/CCR2 axis took place [43]. High protein intake in unilaterally nephrectomized rats further amplified solitary-kidney hyperfiltration: GFR values were the highest early in life but then declined, with severe proteinuria and shortened survival. Protein restriction acts in a protective way and slows the trajectory of GFR decrease [33]. A more recent study on 5/6-nephrectomized mice confirmed this interaction between nephron loss and protein load. High-protein diet aggravated glomerular and tubular injury only in CKD animals, whereas kidneys of sham-operated controls remained structurally intact over a comparable period of follow-up [16]. Developmental models suggest that early-life nutrition may influence susceptibility to later kidney injury. In rats exposed to intrauterine protein restriction, reduced nephron number at birth made the kidney more vulnerable to postnatal stressors. When these animals were subsequently fed a high-protein diet, they developed higher blood pressure and proteinuria, together with structural renal changes. Increased expression of podocyte stress markers further indicated that a high-protein diet may aggravate renal injury programmed by adverse prenatal nutrition.

### 4.2. Structural and Functional Adaptations

Diets providing 30–35% of total energy from protein induced significant increase in renal plasma flow and GFR in rats within weeks. They were followed by glomerular basement membrane thickening, mesangial expansion, and focal segmental glomerulosclerosis with prolonged exposure [43,44]. These effects are partly reversible when dietary protein returns to normal levels but can become fixed if injury progresses beyond the adaptive threshold.

Molecular analyses in rat and mouse models have shown the up-regulation of mTOR and TGF-β pathways, increased expression of profibrotic genes, and enhanced oxidative and endoplasmic reticulum stress. Consistent with human mechanistic data, these changes are attenuated when protein is derived primarily from plant-based sources or when alkali supplementation corrects diet-induced acidosis.

### 4.3. Comparative and Translational Models

Experiments in nephrectomized rats demonstrate that pre-existing nephron loss magnifies susceptibility to protein-induced damage [34]. In contrast, intact animals often maintain renal structural integrity despite transient hyperfiltration, illustrating that previous context, rather than absolute intake, determines the risk.

Limited large-animal data (canine and porcine) confirm these findings but show a slower progression, suggesting differences among species regarding absolute protein load per kg of body weight and renal reserve [45,46].

### 4.4. Limitations of Translational Interpretation

While these models establish biological plausibility, they also face limitations.

First, typical protein intake thresholds differ between humans and animal models, the latter being ten times higher. In healthy adults, high protein intake of >1 g/kg body weight/day is associated with approximately 15% of total energy intake being derived from protein [13]. In studies involving rats, a standard-protein diet provides approximately 20% of energy from protein, whereas a high-protein diet provides even 40% of energy from protein [47]. Moreover, the minimum protein-derived energy required for normal growth, reproduction, and lactation in rats is approximately 17.6% [48].

Second, metabolism differences between species are substantial. Third, a lack of dietary diversity (pure casein/soy isolates) constrains ecological validity.

Moreover, direct translation into human conditions faces the obstacle of a disproportion concerning relative chronicity of intervention in animal experiments versus human data. Therefore, extrapolating thresholds from rodents to humans ought to be carried out cautiously. The experimental data should be treated as supportive evidence rather than hard data confirming findings from human studies.

### 4.5. Integrative Perspective

Taken together, experimental evidence suggests that chronic high protein intake accelerates renal structural injury in models with limited nephron number or existing damage, whereas an adequate or plant-based protein diet minimizes these maladaptive responses.

## 5. Evidence from Adult Studies

The number of studies on dietary protein intake and renal outcomes in numerous populations, tested for many years, seems impressive. However, the quality of data is sometimes insufficient to draw conclusions. Information on racial and ethnic diversity remains limited, and details concerning renal outcomes are not always given. Consequently, despite the large sample sizes and long-term follow-up, the translation of the study results to various populations remains uncertain. The most representative and reliable studies, including those providing data on protein intake value in g/kg/day (where available), are summarized in Table 1.

### 5.1. Observational Evidence in the General Population

Large-scale prospective cohorts examining usual diets suggest that protein source matters for kidney outcomes. The ARIC cohort included almost 12,000 healthy adults (no diabetes, no cardiovascular disease) followed for 24–26 years. Higher intakes of certain animal protein-rich foods were associated with a higher risk of incident CKD stage 3. Contrarily, higher plant protein intake tended to be neutral or favorable after multivariable adjustment [49,53]. Among 3000 citizens of Gubbio (Italy), high protein intake, along with four other modifiable factors, was associated with more significant decline in eGFR in a 15-year follow up [54]. On the contrary, a special RNPC© diet did not trigger a decline in renal function in overweight populations, either with or without CKD [55]. That diet provided 1.5 g/kg/day of protein for men and 1.2 g/kg/day for women during the first phase. In the second phase, the protein content was reduced in such a way that protein accounted for 25% of total energy intake [55]. A meta-analysis summarizing data from 28 studies on adults with DMT2, hypertension, but without CKD, evaluated the estimated glomerular filtration rate (eGFR) before and after the implementation of a high-protein diet (containing >1.5 g/kg/day of protein, providing >20% of energy from protein, or containing more than 100 g of protein) [56]. “Pre-intervention” referred to baseline kidney function assessed prior to initiating the dietary intervention, “post-intervention” concerned the eGFR measured after completion of the high-protein diet period. The duration of dietary intervention among the studies included in this meta-analysis ranged from 4 days to 104 weeks. Results showed that higher protein intake was correlated linearly with a higher eGFR, but there was no connection with change in kidney function before versus after intervention [56].

A cross-sectional study on 6230 adults suggested that dietary patterns characterized by higher overall diet quality and greater intake of plant-based foods were associated with better kidney function, as assessed by the eGFR, and a lower inflammatory burden, compared to diets richer in red and processed meats [31]. The quality of diet was assessed with the Healthy Eating Index, which takes into account, among other factors, the consumption of plant-based protein and seafood. On the other hand, the Healthy Eating Index reflects overall dietary patterns rather than protein quality alone [31]. The red-meat-based diet has been linked to higher microalbuminuria, gut microbiota dysbiosis and even higher mortality among U.S. citizens [57].

### 5.2. Protein Intake and Hard Outcomes in Adults with CKD

Among the elderly with mild-to-moderate CKD, a pooled multicohort analysis reported that higher total protein intake (including both animal and plant sources) was associated with lower all-cause mortality [51]. The analysis suggested that, in that age group, the risk gradually decreased as protein intake increased, with the lowest adjusted risk observed at intakes of around 1.2–1.4 g/kg/day. These data emphasize the need to balance renal protection with sarcopenia prevention in older CKD patients [51]. In analyzed studies, albuminuria represented a surrogate marker of kidney damage rather than a hard outcome. Albuminuria reduction was not an endpoint in the studies discussed in this review. One study specifically assessed the effect of HPD on albuminuria [57], reporting no increase in albuminuria associated with the intervention. Available evidence regarding dietary protein intake and albuminuria are limited.

### 5.3. Progression of CKD and Low-Protein Diets (LPD)

An umbrella review/meta-analysis and prior systematic reviews concluded that LPDs reduced the rate of eGFR decline and the risk of kidney injury, with limited or no impact on all-cause mortality [11]. The LPD, defined as protein intake of 0.6–0.8 g per kg of body mass with >50% of plant-based protein, was associated with slower CKD progression [58]. Moreover, LPD has been associated with higher serum bicarbonate levels, lower phosphorus level, lower azotemia, and a trend towards slower end-stage kidney disease (ESKD) development [59]. However, Pfeiffer et al. stated that patients with diabetes mellitus type 2 and moderate renal impairment (eGFR from 60 to 45 mL/min) could sustain a diet consisting of 0.8 g/kg/d without significant loss of glomerular function [60].

In a randomized controlled trial involving participants with CKD, but without diabetes, there was no difference in eGFR decline between patients with moderate CKD receiving a low protein diet versus those receiving a moderate-protein diet. However, in patients with an eGFR of 13–24 mL/min/1.73 m^2^, a very-low-protein diet supplemented with keto-analogs provided a modest benefit compared with a low-protein diet [61].

Multiple analyses of interventional evidence indicate that protein restriction can slow CKD progression in non-dialysis adults. Despite this, the quantified effects are not uniform, and the level of certainty surrounding the evidence is only moderate to low, stemming from the diverse intake goals, adherence levels, and combined treatments employed. However, other factors hampering objective interpretation of LPD impact on CKD progression should also be taken into account. Multiple comorbidities may trigger low protein intake by adding to poor appetite, declining health status, increasing frailty, medical non-adherence, or low self-esteem among CKD patients. Dietary restrictions in CKD, aggravated by demands concerning intake of high-quality plant-rich diet, may lose out in a clash with insufficient resources due to low socioeconomic status. Therefore, the interpretation of the results discussed above should remain cautious.

### 5.4. Quantity vs. Quality: Animal vs. Plant Protein in CKD

Recent expert reviews conclude that typical targets of protein intake fall around 0.6–0.8 g/kg/d in established non-dialysis CKD patients, with growing emphasis on protein quality. Substituting part of animal protein with plant protein may lower acid load and uremic toxin generation, improve albuminuria, and potentially attenuate progression, provided essential amino acid adequacy is maintained [12]. Hsu et al. proved that a 10% increase in plant-based protein intake diminishes acid load, increases bicarbonate levels and adherence to LPD [62].

The choice of protein source in CKD should also account for nutrients naturally accompanying protein-rich foods—potassium, phosphorus, calcium and fatty acids [63]. Plant-based protein sources including legumes, nuts, and seeds contain substantial amounts of potassium, but its bioavailability from whole plant products is lower than that from animal sources and additives [64]. A similar consideration applies to phosphorus. The plant-derived one is predominantly stored as phytate and is less readily absorbed than that from animal-derived foods or inorganic phosphate additives [63]. As these studies show the absolute potassium and phosphorus content of plant foods, this should not necessarily preclude their use as protein sources in patients with CKD [63,64].

From a practical perspective, minimally processed plant proteins may represent a favorable option for many patients with CKD [64]. Fish also represent an appropriate animal protein source, providing high-quality protein together with long-chain n-3 polyunsaturated fatty acids [65]. Dairy products provide high-quality protein and are an important source of calcium. They also contribute substantial amounts of phosphorus, potassium, and, depending on the product, saturated fatty acids. Their intake should be individualized according to mineral metabolism and overall nutritional requirements [66].

However, planning a diet for a CKD patient must not concentrate on a simple calculation of the number of protein grams consumed per day. Moreover, an even balance and adequate share between plant- and animal-derived protein is not sufficient. Dietary fat and carbohydrate content also have to be taken into account, as they may significantly elevate caloric load. Therefore, since proportions of macronutrients stay relevant for CKD progression, daily caloric demand must be properly divided between protein, carbohydrates and fats.

Although efforts to invent a universal dietary approach seems helpful, CKD specificity (disrupted and progressing acid–base, potassium and calcium–phosphate balances) prevents the unification of recommendations for micronutrients and protein combined intake. Existing guidelines try to systematize instructions, depending on CKD stage, patient’s age and body constitution [67,68]. However, they rely on a variety of suggestions and expert opinions, so strong uniform recommendations are not available. From that perspective, support of dietitians with clinical experience is crucial for the adjustment of a dietary regime and maintaining kidney function in the era of personalized medicine. The latter matters most in the case of CKD patients, a group known for their noncompliance.

Moreover, according to KDOQI/KDIGO guidelines, the evidence on animal versus plant dietary protein effect on nutritional status, calcium–phosphate metabolism or blood lipid profile is insufficient to recommend a particular protein type in CKD patients [67,68]. Explicitly, dietary prescriptions, including those on protein intake, should be personalized in patients with CKD.

### 5.5. Practical Interpretation

Chronic high protein intake can induce hyperfiltration in adults with normal kidney function, but it is not uniformly linked to incident CKD, provided the diet quality is high and plant proteins are predominant. In adults with CKD the balance shifts: modest protein restriction (individualized, dietitian-guided) remains effective in slowing CKD progression. In older CKD populations, a reduced protein intake risks sarcopenia and mortality, whereas prudent higher protein intake may be beneficial. Personalization by age, comorbidity, baseline eGFR, and dietary pattern is crucial [51].

### 5.6. Protein Intake in the Elderly

While analyzing protein demand, the elderly (defined as those over 65 years) appear to be a group of exceptions. The examination of protein intake under age-related longitudinal decline of kidney function (ranging from −0.37 to −1.07 mL/min/1.73 m^2^/year) is to be confronted with the need for adequate diet in order to modulate muscle metabolism [69].

Adults are at increased risk of sarcopenia because the aging body has a reduced ability to maintain muscle mass. Several factors contribute to this process. Insulin sensitivity gradually declines with age, reducing anabolic responses to nutrients. Levels of anabolic hormones, including IGF-1, also decrease over time. In addition, many elderly individuals experience chronic low-grade inflammation related to comorbidities, including CKD. Reduced physical activity, often caused by degenerative musculoskeletal changes, further accelerates muscle loss [70,71,72]. The starting point of losing muscle mass is determined at 30 years of age with a pace of approximately 0.5–1% of its value annually at the age of 50 [73]. As a result, 10% to 27% of people over 70 develops sarcopenia [74].

A low-protein diet (LPD) was suspected to ease sarcopenia development in older patients with CKD. Mafra et al. collected data from various trials yet did not provide arguments to prove that thesis [75]. Surprisingly, one of the trials proved a reduction in body mass, without losing muscle mass, which sounds promising from the perspective of obesity. The evidence suggested that LPD was safe for patients with CKD in terms of a higher risk of developing sarcopenia.

With diabesity being the major causative factor for CKD in adults, GLP-1 receptor agonists may soon become the first-line treatment. This group of patients, together with the elderly, may be at greater risk of GLP-1 receptor agonist–induced sarcopenia, although evidence supporting this association remains limited [76]. Increased protein intake may reduce the risk of muscle mass loss associated with these medications [76,77]. On the other hand, the use of GLP-1 receptor agonists may prevent or lessen sarcopenia in CKD patients, given their anti-inflammatory, antioxidant, and beneficial metabolic effects [78].

Facing common sarcopenia in the elderly, a new possible indicator of life longevity, thigh circumference, has been revealed. Chao-lei Chen et al. came to a conclusion that a wider thigh muscle correlated with a 4% and 6%, lower risk of all-cause mortality and death, respectively, due to cardiovascular background [79]. Generally agreed recommendations for the average adult population to ingest 0.8 g/kg of body mass cover only basic demand sustaining nitrogen balance [80]. Yet, up to 35% of the European elderly living in institutional care units do not reach the level needed to preserve muscle integrity [70].

Having assessed the intricate situation of senior patients, the International PROT-AGE Study Group [69] and European Society for Clinical Nutrition and Metabolism (ESPEN) [70] released the following instructions: 1.0–1.2 g protein/kg/bw for a healthy population over 65 years of age, 1.2–1.5 g protein/kg/bw in the group with acute or chronic illness and 2.0 protein g/kg/bw for the malnourished [70,73]. Consequently, addressing higher amounts of macronutrient may also improve immune system capability, wound healing, and maintaining blood pressure skeletal integrity [72].

Even if those amounts of protein consumed daily do not fulfil the definition of a high-protein diet, exacerbation of underlying kidney dysfunction among the elderly should be reassured. Equations used in the elderly population to estimate kidney function often underestimate nephron reserve in patients with preserved renal function and eGFR values oscillating around 60 mL/min/1.73 m^2^. In this range, the MDRD equation may underestimate kidney function by approximately 25%, whereas the CKD-EPI equation underestimates it by around 16% [70].

In order to practically evaluate the risk of CKD exacerbation in the elderly by administering a protein-abundant diet, Al Qannas et al. reviewed original cohort studies searching for association between protein intake and kidney function in community-dwelling older adults (all over 65 years of age) [81]. Patients included in the review were characterized by a normal kidney function or non-dialysis CKD. Kidney function was assessed by calculating eGFR, using creatinine-, cystatin C- or creatinine–cystatin C-based equations. Albuminuria, as a marker of CKD, was not taken into consideration in any of the following studies.

No significant relation between total protein intake and progression towards CKD was found after 7-year [82] and 12-year follow-ups [83].

Observations made by Beasley et al. seem more reliable due to the use of cystatin C for eGFR calculation. Different clinical scenarios might induce discrepancies between filtration rate assessed based on creatinine versus cystatin C. Creatinine, as a muscle activity metabolite, in sarcopenia might be misleadingly low, overstating renal function [84]. Cystatin C, a small protein produced by all nucleated cells, as a muscle-mass independent factor, provides a relatively reliable and steady measurement over time despite malnutrition or sarcopenia [85,86]. An eGFR decline was observed among patients with a primarily decreased eGFR, although protein restriction was connected with a greater risk of mortality [87]. In one research study also concerning the very elderly (three subgroups of 69–71-, 79–81-, 89–91 years of age), the mean protein ingestion of 1.50 g/kg/day did not result in a lower eGFR. A rapid decline of kidney function in the case of increased protein intake (>16.9% energy) was observed only in those who underwent myocardial infarction. In detail, each redundant protein intake of 0.1 g/kg of ideal body weight resulted in an eGFR–cysC decline of 0.12 mL/min./1.73 m^2^, regardless of the protein source [88]. Collected data suggest that, despite an advanced age and concomitant CKD, elderly patients with high protein intake are more prone to cardiovascular death than progression of renal damage [89].

Low muscle mass is not the only factor that may affect creatinine-based eGFR estimates. In patients with higher levels of physical activity and greater muscle mass, increased creatinine production may result in an underestimation of the eGFR [90]. Similarly, high meat intake and creatine supplementation may increase serum creatinine levels, thereby leading to an underestimation of the eGFR and potentially resulting in an inaccurate diagnosis of actual kidney function [91].

The search for an association between protein source in diet and shifts in eGFR gave inconsistent results across ethnic groups. Sachimi et al. implied that higher animal-derived protein intake in the Japanese cohort resulted in a lower risk of CKD [83]. Contrarily, Caucasian elderly women (average yearly eGFR decline of 0.64 mL/min/1.73 m^2^) presented with a reduction in annual eGFR decline by 0.12 mL/min./1.73 m^2^ with each 10 g of plant-derived protein intake [92]. Animal protein intake did not show such effect. Slower decline rate stayed constant after adjustment for age, BMI, presence of comorbidities (diabetes mellitus, ischemic cardiovascular disease, hypertension, coronary heart disease) [92]. Follow-up thresholds in that study were set for 5 and 10 years of observation.

Although the data were collected precisely, there is still a risk of bias in some of the analyzed studies, especially those based on food quality and dietary pattern questionnaires. Due to the contradictory impact of elevated protein intake in different cohorts and nationalities, there is still a need for further research. Evidently, not only protein alone but also previous lifestyle and overall dietary regimes at different latitudes and in different world regions might determine kidney outcome.

## 6. Pediatric Evidence: Growth Versus Renal Protection

The evidence regarding dietary protein and kidney outcomes in children requires a separate analysis because of multiple age-dependent discrepancies when confronted with adult conditions. Unfortunately, the data linking high protein intake to kidney function in children are limited. Therefore, this section distinguishes between guidelines based on direct evidence from pediatric studies, recommendations derived from pediatric expert opinions and data on kidney outcomes in healthy children and those with chronic kidney disease.

### 6.1. Rationale and Guiding Frameworks

#### 6.1.1. Obesity-Related Consequences in Children with Normal Kidney Function

Dietary trends observed in adults also apply to the pediatric population. Nowadays, Western societies tend to ingest excessive amounts of protein. The average intake in children surpasses recommendations by 1.40–2.39 times in the UK [93] and by 1.88–3.88 times in Spain [94]. Scientists distinguish two main periods in child development, coupled with a significant spike of protein in their diet. The first peak appears at the age of 12 months, when an infant’s diet is converted to cow’s milk, dairy foods and the family usual diet with a higher protein load. The second rise occurs during the preschool period, when more vegetables and whole grains are introduced [95].

Obesity-related nephropathy has become a new challenge in treating the consequences of excessive body mass [96]. Therefore, an increasing number of trials are being designed to get an insight into a possible connection between two self-powered states of hyperfiltration: meals rich in protein in childhood and obesity in adolescence/adulthood [97]. The primary analyzed variables include body mass index (BMI), body mass index standard deviation score (BMI-SDS), the Fat Mass Index (FMI) and Fat-Free Mass Index (FFMI) [98,99,100,101]. Guenther et al. detected a positive association between elevated protein intake in the abovementioned periods with BMI-SDS and body fat percent (BF%) at 7 years, but the endpoint of the research was contradictory. Among toddlers, higher protein intake of animal origin resulted in higher BMI-SDS in the future. Diets rich in protein, presumably plant-derived meals, consumed later in childhood (at 5–6 years), led to an inverse relationship with body fat percentage (BF%). Thus, the impact of excessive supply may vary depending on developmental period and macronutrient composition. A possible explanation for this observation may lie in the physiological mechanism of production of anabolic IGF-1. Some papers reveal that increased protein ingestion promotes rising IGF-1 concentrations [99,102]. Other studies conclude there is a positive connection between high protein intake and increased FFMI [97].

Those observations concern associations between growth, body composition, and adiposity yet neglect long-term kidney outcomes. In consequence, direct pediatric evidence linking high protein intake with transient kidney damage in otherwise healthy children is scarce [103]. Evidence on a potential transition to persistent renal injury is insufficient either. Therefore, any extrapolation from adult evidence should be cautious.

#### 6.1.2. Children with CKD

Protein intake in children with CKD must support linear growth and neurodevelopment, while avoiding maladaptive hyperfiltration and excess acid/phosphate load. Contemporary care in pediatrics draws heavily on the Pediatric Renal Nutrition Taskforce (PRNT) clinical practice recommendations, which synthesize evidence across CKD stages 2–5 and dialysis and emphasize individualized targets, frequent monitoring, and dietitian-led adjustments [104]. In brief, the PRNT advises one to align protein intake with age-specific needs and modestly increase it on dialysis to cover dialytic losses, while avoiding excessive protein load in pre-dialysis CKD.

Current pediatric recommendations should be distinguished from direct evidence that protein restriction improves renal outcomes in children. Pediatric outcome data remain limited to contemporary reviews [105]. They do not support routine protein restriction as a strategy to slow CKD progression in this population. On the contrary, inadequate protein intake may increase the risk of malnutrition and protein-energy wasting [105].

A recent educational review integrates all PRNT recommendations for children aged 1–18 years, underscoring practical implementation (meal planning, label reading, mitigation of phosphorus additives) and the challenges aggravating with CKD progression and increased intake of processed foods [106].

### 6.2. Age-Related Conditions

#### 6.2.1. Infancy and Early Childhood (0–2 Years)

Infants with CKD require relatively higher protein supplementation per kilogram than older children to sustain rapid linear and brain growth. When oral intake is insufficient, fortified formulas or enteral nutrition is necessary [67,106]. The PRNT infant-focused guidance details strategies for adjusting energy and protein density, managing metabolic acidosis, and balancing mineral intake. Observational pediatric data also warn that higher protein intake without adequate alkalization may correlate with lower plasma bicarbonate and impaired bone mineral metrics, highlighting the need for acid–base monitoring.

#### 6.2.2. School-Aged Children and Adolescents (2–18 Years)

Nutrient prescriptions for children and adolescents should be recalibrated with growth velocity, CKD stage, and comorbidities (e.g., acidosis, anemia, CKD–MBD). Reviews emphasize avoiding chronic protein overfeeding in non-dialysis CKD, while ensuring adequacy for growth. On chronic dialysis, staged advancements cover dialytic amino acid losses, with special focus on method-related protein losses in children on peritoneal dialysis. Education on protein quality and phosphate additives (processed meat/cheese) is central to concomitant protein goal achievement and CKD–MBD avoidance [106].

A scoping review of pediatric dietary intake underscores heterogeneity and gaps in reported nutrient intake (including protein) and identifies subgroups with suboptimal or poorly described intake patterns—supporting PRNT’s call for individualized assessment rather than one-size-fits-all targets [107].

### 6.3. Protein Source and Metabolic Milieu

Consistent with adult data, pediatric guidance increasingly promotes a greater share of plant protein when feasible. Reduced dietary acid load and phosphate bioavailability are recommended, provided total indispensable amino acid needs are met. Practical tactics includes replacing red/processed meat with legumes, soy products, eggs/dairy in moderation, and emphasizing minimally processed options to limit inorganic phosphate additives [29].

### 6.4. Alignment with Broader CKD Nutrition Guidance

General CKD nutrition guidelines [108] reinforce principles of assessment, monitoring, and tailored protein prescriptions by current kidney function and clinical status. Pediatric teams commonly adapt these adult frameworks to the higher growth demands and unique feeding issues of children (Table 2).

## 7. Clinical Implications and Recommendations

### 7.1. Balancing Nutritional Needs and Renal Protection

Findings from mechanistic, clinical, and epidemiologic studies converge on the need to balance nutritional adequacy with renal workload. In CKD, this means tailoring protein intake to disease stage and comorbidities, while avoiding both excess restriction (malnutrition, sarcopenia) and chronic overload (hyperfiltration, acidosis) [8,12,109]. For non-dialysis adults with CKD, current guidelines (KDOQI 2020 [108]; ESPEN 2021 [68]) recommend 0.6–0.8 g/kg/day, suggesting a significant share of plant-based sources. For dialysis patients, increased targets (up to 1.0–1.2 g/kg/day) should compensate for dialytic amino acid losses. Irrespective of CKD stage, all metabolically stable adults are to be provided with adequate energy intake of 25–35 kcal/kg body weight/day, based on age, sex, level of physical activity, body composition, concurrent illness or presence of inflammation, to maintain normal nutritional status [108]. For children, the Pediatric Renal Nutrition Taskforce (PRNT) advises individualized age-adjusted intakes, with higher per kg needs in infants and modest increments on dialysis [68,108,110].

Table 3 provides guidelines regarding protein intake across different age groups, taking into account CKD stage, the presence of diabetes, and metabolic status.

It is worth mentioning that among multiple nutritional guidelines for CKD patients, the only recommendations with high-quality evidence (1A) concern protein restriction to 0.55–0.6 g/kg/day in metabolically stable non-diabetics with CKD stages 3–5, in order to reduce the risk for end-stage kidney disease and death [108]. Other guidelines’ quality is usually limited to low-evidence suggestions (2C) or opinions [108,111].

### 7.2. Protein Quality and Source

Substituting animals with plant protein provides multiple advantages: lower phosphorus bioavailability, reduced acid load, greater fiber intake, and potential improvement in metabolic parameters. Importantly, combining plant and animal proteins (e.g., eggs, dairy, legumes) ensures essential amino acid adequacy while leveraging the metabolic benefits of plant sources [28,114].

Longitudinal pediatric data are scarce. Most recommendations remain extrapolated from adult or expert consensus. The optimal mix of plant versus animal protein in early CKD remains undefined, particularly across diverse diets and cultural patterns. Interventional trials combining diet modification with pharmacologic nephroprotection (e.g., SGLT2 inhibitors) are warranted. The interplay between microbiome composition and nitrogen metabolite handling under varying protein intakes is an emerging field requiring attention.

## 8. Primary Care Restrictions and Community Challenges

Facing patients on a high-protein diet in primary care conditions means estimating their kidney function based on serum creatinine level, an imperfect indicator dependent on total muscle mass, individual dietary strategy, supplement/creatine intake, malnutrition, or age [115,116]. What needs to be emphasized is that laboratory measurement value does not rise linearly alongside kidney filtration decline. Namely, if creatinine serum level of 1 mg/dL equals one’s normal kidney function, a rise to 2 mg/dL, 4 mg/dL, or 8 mg/dL corresponds respectively with a 50%, 70% to 85% and 90% to 95% reduction in eGFR [117]. Thus, the progressive nephron loss, balanced by the remaining nephrons’ hypertrophy and hyperfiltration, is overlooked in routine blood tests before the irreversible burden occurs. Statistics reveal that one in seven U.S. citizens suffer from CKD and nine out of 10 remain unaware of the condition [118]. Moreover, up to 82% of people with CKD stage 3 and half of the group with CKD stages 4–5 remain undiagnosed [119].

Additionally, using only serum creatinine to calculate GFR values leads to an overestimation of kidney function due to creatinine filtration by glomerulus and secretion by the tubules [28,120]. Tubular secretion simultaneously increases CKD progression and glomerular filtration decline, leading to eGFR overprediction by 10 to 60% [121,122]. In 2020, Oba et al. tried to estimate a single-nephron filtration rate, dividing corrected serum creatinine clearance by the number of non-sclerotized glomeruli. Means required to establish a total nephron number included cortical volume and glomerular density, significantly more advanced, and imaging-derived procedures unavailable in ambulatory care circumstances [28].

Another crucial aspect in a discussion about high-protein diets is that their deleterious effect focuses not only on kidneys, but it affects the whole organism homeostasis. Opposite effects mentioned in the literature include decreased control of blood pressure (BP) and blood glucose level or glucose disposal rate in patients with detected renal hyperfiltration despite intensive treatment [123].

Moreover, high-protein dietary strategy strongly and positively corresponds with phosphorus load. According to Boaz and Smetan [124], rich-in-protein food ingestion may be responsible for 84% of the variance in phosphorus consumption [125]. In a prospective cohort study of healthy US adults, Chang et al. concluded that excessive phosphorus ingestion, defined as 1400 mg/d, correlated with higher all-cause mortality. That underlines phosphorus overload as another public health problem, while as much as one-third of Americans report surpassing that threshold in their dietary pattern [126]. Simultaneously, hyperphosphatemia remains the most prevalent mineral abnormality, affecting approximately 50% of the patients on hemodialysis [127,128]. Furthermore, 19 to 57% of this population also struggles to adhere to a phosphorus- restrictive diet [127,129]. What requires more insight when planning an eating strategy is the source of phosphorus in the diet (plant-derived vs. animal-contained). Needless to say, highly processed food forms an instant easily accessible source of phosphate in today’s ready-to-use commercial massive cuisine.

Therefore, individual decisions to set a high-protein diet as a basic dietary strategy with unknown clinical kidney status or without medical supervision remain potentially detrimental.

## 9. Summary

Dietary protein intake is essential for kidney health, conditioning renal growth, tissue repair, and metabolic integrity. Experimental data and animal studies confirm that renal response to short-time high protein load results in an adaptive increase in intraglomerular pressure and subsequent hyperfiltration, yet without proof of negative effects on a healthy kidney. Moreover, the long-term effect of chronic kidney exposure to excess protein intake also remains uncertain.

The direct translation from animal experiments seems risky due to essential inter-species differences in protein and calorie demands, various absolute values of high-protein threshold, or disproportion in relative chronicity of intervention in animal experiments versus human data.

The multiplicity of human studies, analyzing protein impact on kidney function, does not translate into strong recommendations either. Variable methodologies (prioritizing observational over interventional studies, with common questionnaire-based evaluations), basic kidney outcomes (mainly creatinine-based eGFR), unequivocal interest in age groups (priority to the elderly at the cost of pediatric patients), or frequent neglect of confounding factors (protein intake analyzed without dietary/medical/socioeconomic/comorbid background), result in conclusions on the preference of a plant over an animal source of protein but stressing individual approaches.

Taking into account disparities in dietary patterns among geographical regions and ethnicities, it is impossible to unify guidelines concerning appropriate protein intake, ensuring kidney health, metabolic integrity and satisfactory quality of life. However, even a sketchy confrontation of Western societies, with relatively high chronic protein intake and a shortening lifespan due to comorbidities including diabesity, versus Eastern populations, characterized by plant-based dietary patterns and longevity, should raise deep concern and trigger long-term comparative multi-ethnic studies.

Thankfully, the strongest evidence concerns those who may benefit the most, patients with chronic kidney disease. Experimental data prove that previous renal injury may aggravate in the course of chronic protein overload, turning adaptive hyperfiltration into maladaptive restriction of renal functional reserve and faster progression of CKD. Evidence from large cohorts and meta-analyses in adults supports moderate protein restriction to slow CKD progression, with emphasis on protein quality and source than on quantity alone.

However, even if substitution of animal proteins with plant-based alternatives is increasingly recognized as a viable strategy to reduce metabolic acid load, phosphate exposure, and uremic toxin burden, no evidence justifies the preference of a particular source of protein in CKD patients.

In pediatric CKD, the paradigm shifts toward supporting normal growth with simultaneous prevention of hyperfiltration. Guidelines provide detailed CKD stage- and age-specific targets, stressing individualized prescriptions, close monitoring, and professional dietary supervision, although strong evidence is lacking.

Currently, neither universal protein restriction nor chronic high protein intake is appropriate for all CKD patients. Instead, a personalized and stage-specific dietary approach, integrated with medical therapy and regular nutritional assessment, offers the most balanced strategy for preserving renal function.

Future studies—particularly longitudinal pediatric trials and interventions combining dietary modulation with pharmacologic nephroprotection—are essential to refine protein recommendations across CKD phenotypes and life stages. Until such data are available, clinical decisions should prioritize individual needs, current renal functional reserve, and metabolic context, guided by experienced dietitians and multidisciplinary care teams.

## Figures and Tables

**Figure 1:**
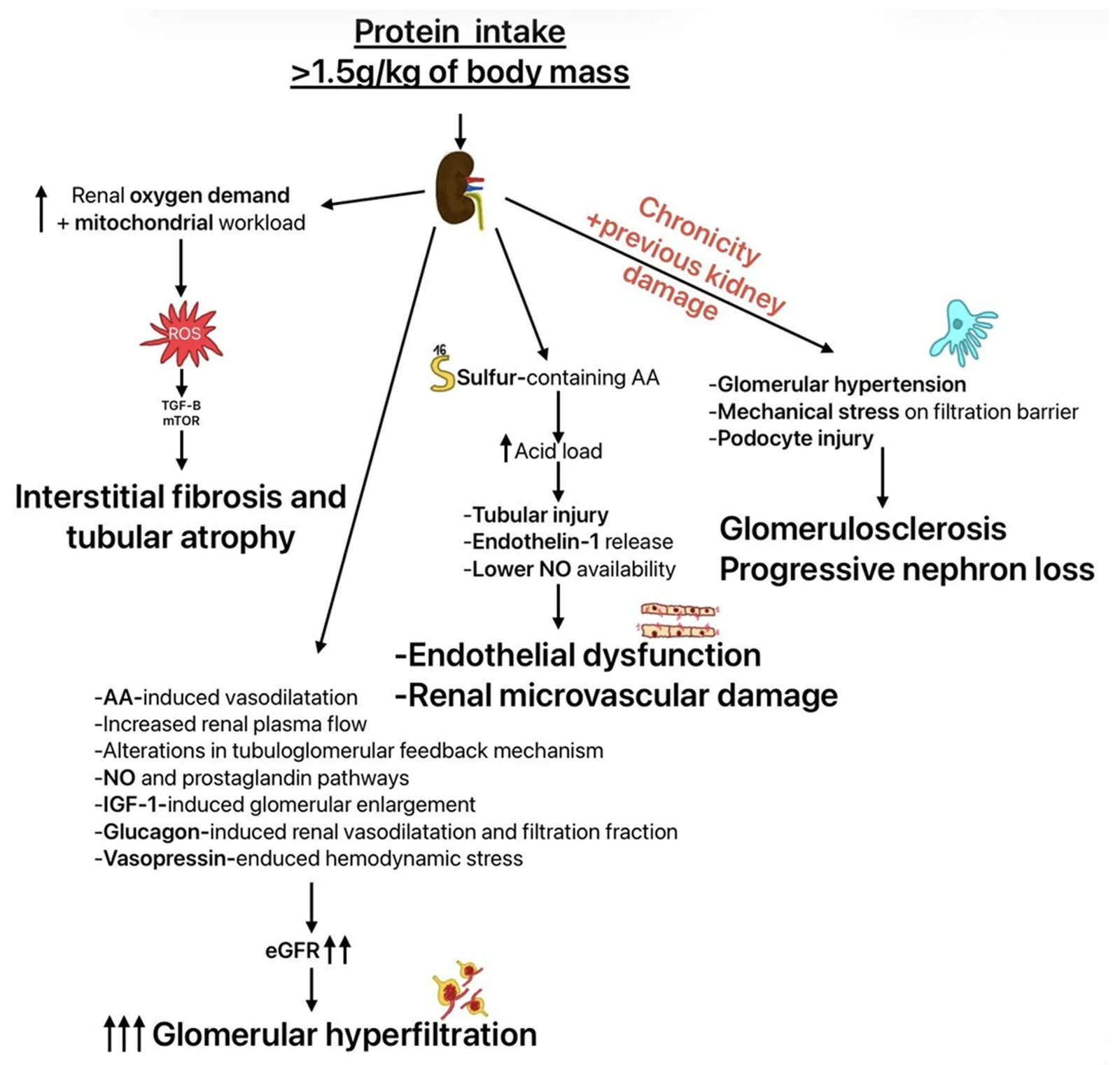
Pathomechanism of excessive protein intake impact on the kidney—created by the authors, based on data from Section 3.1, Section 3.2, Section 3.3, Section 3.4 and Section 3.5 (AA—amino acids; NO—nitrogen oxide; IGF-1—insulin-like growth factor-1; eGFR—estimated glomerular filtration rate; ROS—reactive oxygen species; TGF-beta—tumor growth factor-beta).

**Table 1:** Large-scale studies and meta-analyses on dietary protein intake and renal outcomes in adults.

Study (Year)	Haring et al., 2017 [49]	Hu et al., 2019 [50]	Carballo-Casla et al., 2024 [51]	Yan et al., 2018 [52]	Khosroshahi et al., 2024 [11]	Fouque, 2025 [12]
Design/N	Prospective ARIC cohort; n = 11,952	Prospective ARIC cohort; n = 14,686	Multicohort observational study; n = 8543; 14,399 observations	Systematic review/meta-analysis; 19 RCTs; n = 2492	Umbrella review; 25 meta-analyses including 47 RCTs	Narrative/critical review
Age (years)	44–66	Middle-aged adults	≥60; CKD observations: 78.0 ± 7.2	Mean 51	Not reported as a single pooled age	Not applicable
Baseline eGFR/renal status	eGFR ≥ 60 mL/min/1.73 m^2^; free of diabetes and CVD at baseline; eGFR calculated based on creatinine level	Baseline kidney function considered; study evaluated incident CKD in a community cohort; eGFR calculated based on creatinine level	CKD stages 1–3; predominantly stage 3a; advanced CKD/KRT excluded; CKD stages 1–3; creatinine-based eGFR	CKD; median baseline eGFR 33.5 (range 14.4–96.3) mL/min/1.73 m^2^; assessment methods varied across RCTs	CKD populations of varying severity	CKD; focus on nutritional management, primarily nondialysis CKD
Protein exposure (g/kg/d or study-specific definition)	Total, animal and vegetable protein assessed in quintiles; g/kg/d not reported	Not defined in g/kg/d; exposure based on dietary-pattern scores	CKD: total 1.15 ± 0.37; animal 0.78 ± 0.29; plant 0.37 ± 0.14 g/kg/d	LPD/VLPD 0.29–0.90 g/kg/d; intervention-control difference 0.2–1.1 g/kg/d	Variable LPD definitions across included evidence; no single g/kg/d exposure applicable	Discusses protein quantity and quality and protein-restriction strategies; no single experimental exposure
Dietary assessment	Interviewer-administered validated 66-item FFQ at visits 1 and 3; cumulative average intake	FFQ-based dietary assessment	Validated dietary histories and FFQs	Prescribed dietary interventions; assessment/adherence methods varied by RCT	Umbrella review	Narrative review
Follow-up	Median 23 y	Median ~24 y	Mortality assessed up to 10 y	Median 55 weeks (range: 26–380 weeks)	Variable	Not applicable
Main outcome	Incident CKD	Incident CKD; kidney-function decline	All-cause mortality	Kidney failure; ESRD; eGFR; mortality	Multiple kidney-related outcomes	Clinical synthesis
Adjusted effect estimate (95% CI)	Vegetable protein Q5 vs. Q1: HR 0.76 (95% CI 0.64–0.91); red/processed meat Q5 vs. Q1: HR 1.23 (1.06–1.42)	Pattern-specific adjusted HRs reported in the source publication; no protein-specific HR	Total protein: HR 0.92 (95% CI 0.86–0.98) per +0.20 g/kg/d	Kidney failure: OR 0.59 (0.41–0.85); ESRD: OR 0.64 (0.43–0.96); eGFR decline: MD 1.85 mL/min/1.73 m^2^/y (0.77–2.93)	Outcome-specific pooled estimates varied; certainty of evidence ranged from moderate to very low	Not applicable—narrative review
Key covariates	Age, race-center, sex, education, energy intake, lipids, BP/medications, alcohol, smoking, physical activity, carbohydrate intake, BMI, WHR	Age, sex, race-center, energy intake, education/income, baseline eGFR, physical activity, smoking, BMI, diabetes, SBP, antihypertensive medication, HDL	Demographic, lifestyle, clinical and dietary variables	mMeta-analysis of RCTs	Umbrella review	Not applicable
Study-specific limitations	Observational design; self-reported diet; residual confounding; associations do not establish causality	Observational design; FFQ measurement error; residual confounding; dietary-pattern study rather than quantitative protein-exposure study	Observational design; residual confounding; mortality rather than CKD progression; limited applicability to advanced CKD	Heterogeneous protein targets, CKD severity and interventions; some trials included keto-acid supplementation	Heterogeneous interventions/outcomes; secondary evidence; possible overlap of primary studies; variable certainty	Narrative secondary evidence; no pooled effect estimate; recommendations require individualization

**Table 2:** Key references on dietary guidelines for CKD children.

Evidence Level/Strength	Type of Recommendation	Protein Recommendation/Key Points for Practice	Population/Focus	Source/Year
Upper end of SDI: Level C, moderate recommendation; not below lower end of SDI: Level X, strong recommendation; additional protein on dialysis: Level C, weak recommendation	Target intake/minimum safe intake/increased requirement on dialysis	Protein intake should be maintained at the upper end of the Suggested Dietary Intake (SDI) to support optimal growth. Intake should not be reduced below the lower end of the SDI. Children receiving dialysis may require additional protein to compensate for dialysate losses.	Children with CKD stages 2–5 and on dialysis	Pediatric Renal Nutrition Taskforce (PRNT), 2020 [104]
Clinical practice review based on PRNT recommendations; no independent formal evidence grade assigned	Target intake/nutritional adequacy; not protein restriction	Adequate age-specific protein intake is required to support rapid growth and development. Fortification or enteral feeding may be necessary when nutritional requirements cannot be achieved orally. Protein intake should be individualized according to growth, nutritional status, CKD stage, and dialysis-related losses.	Infants with CKD stages 2–5 and on dialysis	Shaw et al., 2023 [67]
Educational review integrating PRNT clinical practice recommendations; no independent formal evidence grade assigned	Target intake/individualized nutritional prescription/increased requirement on dialysis	Age-appropriate protein intake should support growth and nutritional status, with individualized dietary assessment and attention to protein quality. Additional protein may be required in children receiving dialysis because of dialysate losses.	Children and adolescents aged 1–18 years with CKD and on dialysis	Shaw et al., 2025 [67]
GRADE-based guideline; strength varies by recommendation. Protein restriction in metabolically stable adults with CKD stages 3–5 without diabetes: 1A.	Therapeutic protein restriction/target intake in adults; not a pediatric protein recommendation	Provides individualized protein prescriptions according to CKD stage and treatment. Protein restriction is recommended for selected metabolically stable adults with non-dialysis CKD; these restriction targets should not be directly extrapolated to children because of pediatric growth requirements.	Adults with CKD; included as a broader CKD nutrition framework	KDOQI Clinical Practice Guideline for Nutrition in CKD, 2020 [108]

**Table 3:** Core guidelines for dietary protein intake in the CKD population.

Age Group	Source	Population	Suggested/Recommended Protein
			Intake [g/kg/day]
Adults	KDOQI 2020 [108]	CKD stage 3–5, metabolically stable, without diabetes, not on dialysis	0.55–0.6 (LPD) or 0.28–0.43 with keto acid analogs/amino acid supplementation to meet protein requirements of 0.55–0.6 (VLPD); under close supervision
		CKD stage 3–5 with diabetes, not on dialysis	0.6–0.8
		CKD stage 5D on HD or PD, metabolically stable, with or without diabetes	1–1.2
	KDIGO 2024 [111]	CKD stage 3–5, independently of diabetes diagnosis, metabolically stable	0.8
			avoid >1.3
		CKD stage 3–5 at risk of kidney failure, metabolically stable	0.3–0.4 with keto acid analogs/amino acid supplementation (up to 0.6 g/kg/d) under close supervision
		CKD, metabolically unstable	Avoid low-protein diet
Elderly	PROT-AGE study group [69]	Healthy older adults	1–1.2
		Older adults with acute or chronic disease, except from those with severe kidney disease (eGFR < 30 mL/min/1.73 m^2^)	1.2–1.5;
			up to 2.0 for people with severe illness, injury or malnutrition
		Older adults with severe CKD (GFR < 30 mL/min/1.73 m^2^)	<0.8
		Older adults with moderate CKD (GFR 31–60 mL/min/1.73 m^2^)	>0.8
			eGFR should be monitored 2×/year
		Older adults with mild CKD (GFR > 60 mL/min/1.73 m^2^)	Increase protein intake if needed
		Older adults on PD or HD	1.2–1.5
	ESPEN/ERN-ERA joint review [112]	Older adults with CKD	Personalized approach
Children	PRNT [104]	Children with CKD stage 2–5D, not on dialysis	Age-adjusted per PRNT tables: upper end of the SDI
		Children with CKD stage 2–5D, on dialysis	Higher than SDI
		Children with CKD stage 2–5D and persistently high blood urea levels	Lower end of the SDI
	KDOQI 2008 [113]	Children with CDK stage 3	100–140% DRI for ideal body weight
		Children with CDK stage 4–5	100–120% of DRI for ideal body weight
		Children on PD	100% of DRI + 0.15–0.3 g/kg/day
		Children on HD	100% of DRI + 0.1 g/kg/day
	KDIGO 2024 [111]	Children with CKD stage 2–5	Upper end of the normal range for healthy children; do not reduce protein intake

## Data Availability

No new data were created or analyzed in this study. Data sharing is not applicable to this article.

## Abbreviations

The following abbreviations are used in this manuscript:

AA	Amino Acids
AKI	Acute Kidney Injury
ApoB	Apolipoprotein B
ARIC	Atherosclerosis Risk in Communities
ARRIVE	Animal Research: Reporting of In Vivo Experiments
BF%	Body Fat Percentage
BMI	Body Mass Index
BMI-SDS	Body Mass Index Standard Deviation Score
BP	Blood Pressure
CCR2	C-C Chemokine Receptor Type 2
CKD	Chronic Kidney Disease
CKD-EPI	Chronic Kidney Disease Epidemiology Collaboration
CKD-MBD	Chronic Kidney Disease–Mineral and Bone Disorder
CPRs	Clinical Practice Recommendations
eGFR	Estimated Glomerular Filtration Rate
ESPEN	European Society for Clinical Nutrition and Metabolism
ESKD	End-Stage Kidney Disease
FFMI	Fat-Free Mass Index
FMI	Fat Mass Index
GFR	Glomerular Filtration Rate
HPD	High-Protein Diet
IGF-1	Insulin-like Growth Factor 1
KDOQI	Kidney Disease Outcomes Quality Initiative
LDL	Low-Density Lipoprotein
LPD	Low-Protein Diet
MCP-1	Monocyte Chemoattractant Protein-1
MDRD	Modification of Diet in Renal Disease
mTOR	Mechanistic Target of Rapamycin
NHANES III	National Health and Nutrition Examination Survey III
NO	Nitric Oxide
PRNT	Pediatric Renal Nutrition Taskforce
RCT	Randomized Controlled Trial
ROS	Reactive Oxygen Species
SGLT2	Sodium-Glucose Cotransporter 2
TGF-β	Transforming Growth Factor Beta
TGF-β1	Transforming Growth Factor Beta 1
UK	United Kingdom
US	United States
VLPD	Very-Low-Protein Diet
